# Pinhole Engineering based Enhanced Resolution (PEER) for Fluorescence Lifetime Imaging Microscopy

**DOI:** 10.21203/rs.3.rs-6306076/v1

**Published:** 2025-09-30

**Authors:** Wonsang Hwang, Sinyoung Jeong, J. Matthew Dubach, Conor L. Evans, Iván Coto Hernández

**Affiliations:** 1 Wellman Center for Photomedicine, Harvard Medical School, Massachusetts General Hospital, CNY149, 13th St, Charlestown, 100190, MA, USA.; 2 Intek Scientific, 1 Broadway, Cambridge, 02142, MA, USA.; 3 Institute for Innovation in Imaging, Massachusetts General Hospital and Harvard Medical School, 149 13th St, Charlestown, 02129, MA, USA.

**Keywords:** FLIM, Super-resolution, STED, Confocal

## Abstract

In this article, we present a differential confocal microscopy technique based on pinhole engineering that significantly enhances both lateral resolution and axial sectioning depth. Through simulations and experimental validation with a fluorescent calibration slide, we demonstrated a 1.6-fold improvement in lateral resolution and a two-fold increase in axial sectioning capability. Building on these advancements, we integrated an intensity-weighted lifetime imaging strategy to surpass the diffraction limit in fluorescence lifetime measurements. This approach achieved high spatial resolution and quantitative lifetime data comparable to leading super-resolution FLIM (Fluorescence Lifetime Imaging Microscopy) techniques, yet allows for simpler implementation. We further validated the method in subcellular structure lifetime imaging, demonstrating improved resolution and axial depth enhanced lifetime-based multiplexing capacity. This new method provides an accessible route to high-resolution, multiplexed FLIM for advanced biological imaging.

## 1 Introduction

Confocal fluorescence microscopy is a widely used imaging technique for acquiring high-contrast biomedical images using a physical pinhole that reduces the out-of-focus fluorescent background. A pinhole size of 1 Airy Unit (AU) is generally considered a good trade-off between image intensity and optical sectioning. The effective lateral resolution of confocal images can be further improved by reducing the pinhole size, although this comes at the cost of signal loss and increased shot-noise [1]. Small pinhole sizes between 0.6 and 0.2 AU have been used in bright samples with highly sensitive detectors featuring low background noise, in combination with image deconvolution, to further enhance the signal-to-noise ratio (SNR) and achieve effective resolutions of 140 and 120 nm, respectively [2].

Lateral and axial resolution enhancement in confocal fluorescence microscopy has been achieved by engineering the illumination and detection point spread function (PSF) [3]. Multiple differential confocal microscopy (DCM) techniques have been used as an alternative to improve image quality for both fluorescent and non-fluorescent samples. For example, both the lateral resolution and optical sectioning capabilities in confocal microscopy have been enhanced by subtracting images taken with different pinhole sizes [4]. This approach can be achieved by either simultaneously splitting the signal into two detection channels or rapidly tuning the pinhole size using a motorized pinhole sequentially. However, a key limitation of these subtracting approaches is the increased noise level and loss of information caused by negative values during subtraction, which are clipped to zero, potentially resulting in the generation of image artifacts. Complicating matters is that the amplitude of negative values depends on the pinhole sizes and the system’s magnification [4]. A correction factor has been used to compensate for the differences in intensity between the two images, thus reducing the negative values [4]. For example, Sánchez-Ortiga et al. used a correction factor that makes the value of the open-pinhole image intensity equal to one-half of the value acquired with the closed pinhole [5]. Recently, a phasor-based separation algorithm has been applied to multiple confocal images acquired at different pinhole aperture sizes to improve optical sectioning while preserving the same level of SNR [6, 7]. A CCD camera, as a detector, was adopted to simultaneously collect signals that emulated an open and closed pinhole at an early stage of technological development [5]. The simplicity of this method enables the reconstruction of the subtractive image during acquisition. The recent development of fast single-photon detector arrays and image scanning microscopy reconstruction algorithms have been used to further improve optical sectioning and discrimination between foreground and background [8, 9]. However, the use of these array detectors, which typically have between 25 and 49 sensors, imposes limitations on both the image acquisition card’s ability to handle signals from multiple sensor elements and the software’s capacity to efficiently process all the data for real-time image reconstruction.

Over the last three decades, multiple super-resolution (SR) microscopy techniques based on different physical principles have been developed to break the diffraction barrier in the far field. Stimulated Emission Depletion (STED) microscopy can achieve a resolution of up to 30 nm in biomedical samples, allowing advancements in biomedical investigations at the nanometer scale [10]. A subtraction microscopy technique called fluorescence emission difference (FED) improves this approach by reducing the high illumination power used on STED microscopy [11]. This technique is based on the intensity difference between two microscopy images collected with the same excitation wavelength, but using two different illumination beams (Gaussian- and donut-shaped wavefronts). Recently, computational techniques have been developed to overcome the complexity and high cost of these imaging systems, making them more widely accessible. SR images has been achieved within a single fluorescence image using deblurring by pixel reassignment (DPR) [12] and super-resolution methods based on mean-shift theory (MSSR) [13]. However, the complexity of these algorithms has limited their rapid and widespread adoption.

Multimodal super-resolution imaging has been achieved by combining super-resolution microscopy with other imaging techniques, providing complementary information from the sample and/or improving spatial resolution[14]. The combination of fluorescence lifetime imaging microscopy (FLIM) with super-resolution enables better separation of overlapping spectral fluorescence signals from nearby biological structures, improving the ability to resolve fine details. This combination has been reported in several configurations, such as STED and image scanning microscopy [15, 16]. In this article, we propose a differential, time-resolved confocal microscopy technique called Pinhole Engineering-based Enhanced Resolution (PEER), which is based on the dual subtraction of two time-resolved confocal images acquired with different pinhole sizes. First, an image similar to those obtained by scanning the sample with a donut beam is generated, followed by the reconstruction of a super-resolution image that surpasses the resolution of confocal and DCM techniques. Through the simulations and experimental validation using a fluorescent calibration slide, we verified a 1.6-fold improvement in lateral resolution and a two-fold increase in axial sectioning depth capability. Finally, an intensity-weighted imaging strategy, similar to those employed to enhance image visualization in fluorescence lifetime [17] and anisotropy imaging [18], was used to improve the resolution of confocal fluorescence lifetime imaging and support multiplexing. Intensity-weighted super-resolution fluorescence lifetime images were computed on biological samples to provide nanoscale spatial and lifetime information, similar to STED-FLIM and ISM-FLIM, but with a simpler implementation.

## 2 Results

### 2.1 Lateral resolution enhancement by pinhole engineering

We consider confocal images of a centered point source. The images were acquired using both an open and a closed pinhole. The image acquired with a closed pinhole $I_{CP}(x,y)$ is scaled by a correction factor α and then subtracted from the image acquired with an open pinhole $I_{OP}(x,y)$ to yield a donut-shaped image:

(1) $$I_{D}(x,y)=I_{OP}(x,y)-\alpha \cdot I_{CP}(x,y),$$

In what follows, $I_{OP}(x,y)$ and $I_{CP}(x,y)$ represent confocal images captured with pinhole sizes of 2 AU and 0.5 AU, respectively, and α is chosen to compensate for differences in signal intensity.

The SR image is subsequently reconstructed by subtracting a scaled version of the donut image (i.e., β·Donut) from the image acquired with a closed pinhole $I_{CP}(x,y)$:

(2) $$I_{SR}(x,y)=I_{CP}(x,y)-\beta \cdot I_{D}(x,y).$$

Here, β is a weighting factor that governs how much of the components in the SR image compared to the confocal images (the donut signal) is used during SR reconstruction. The two equations can be combined into one, allowing super-resolution images to be obtained in a single step as a linear combination of the two confocal images. The equation(2) can be rewritten as:

(3) $$I_{SR}(x,y)=I_{CP}(x,y)\cdot (1+\alpha \cdot \beta )-\beta \cdot I_{OP}(x,y).$$

To maintain a trade-off between SNR and resolution enhancement, in the case of low signal, $I_{CP}(x,y)$ in equation (2) can be replaced by $I_{OP}(x,y)$.

**Fig. 1:**
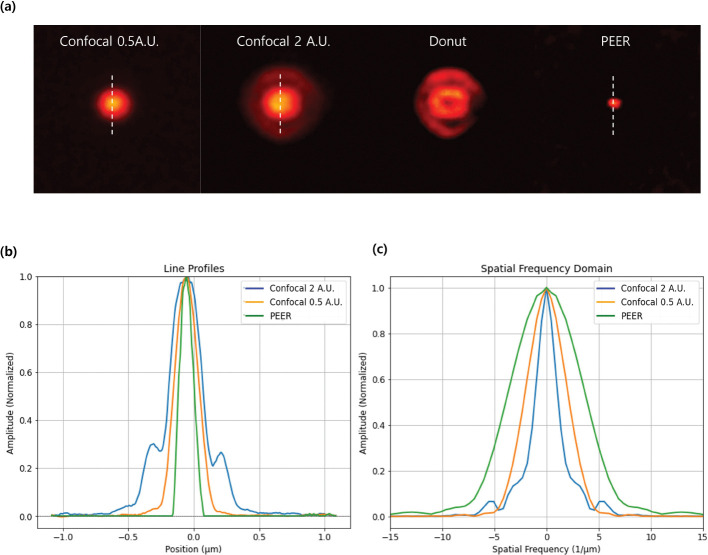
(a) Confocal fluorescence images of 40 nm beads (excited at 640 nm) acquired with pinhole sizes of 0.5 AU and 2 AU. The donut image is generated by subtracting the scaled 0.5 AU image from the 2 AU image. (b) Line profiles along the bead’s central axis for the 0.5 AU image, 2 AU image, and the SR reconstruction, illustrating the improved resolution achieved by the PEER method. (c) Spatial frequency analysis of the line profiles, highlighting the extended high-frequency.

Figure 1 illustrates this method using confocal fluorescence images of 40 nm beads excited at 640 nm. Figure 1(a) shows the confocal images captured at 0.5 AU and 2 AU, the resulting donut image generated by the subtraction process, and the obtained SR image. Figure 1(b) presents the line profiles along the central axis of a bead, highlighting the improved lateral resolution offered by the SR approach compared to the individual confocal images. The sharper and more distinct peaks in the SR line profile reflect better separation between closely spaced beads. Figure 1(c) displays the spatial frequency spectra of these line profiles, where the SR method reveals higher-frequency components than the confocal images. This enhanced frequency range underpins the improved resolution, indicating that the method effectively leverages the additional spatial information contained in the donut image. Overall, these results demonstrate that the new SR method enhances lateral resolution without using additional hardware or complex computational algorithms. By appropriately scaling and subtracting confocal images, the method captures finer structural details, offering a cost-effective and robust approach for high-resolution fluorescence imaging.

**Fig. 2:**
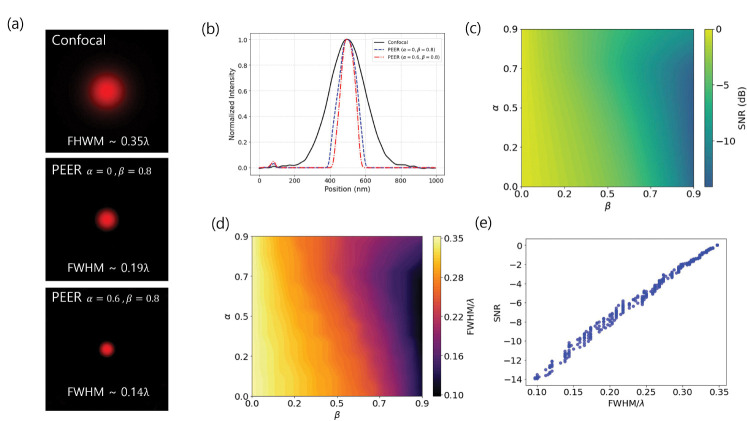
Scaling factor study. (a) Simulated point spread functions (PSFs) for a confocal (0.5 AU) setup and two super-resolution settings (α=0, β=0.8) and (α=0.6, β=0.8). (b) Cross-sectional intensity profiles of the PSFs. (c) A 2D map of the SNR (in dB). (d) A 2D map of FWHM/λ. (e) A scatter plot of SNR versus FWHM*/λ*.

As shown in Figure 2, the scaling factors α and β affect both resolution and SNR in the numerical simulations. In Figure 2(a), we compare PSFs under three configurations: a 0.5 AU confocal baseline and two super-resolution conditions (α=0, β=0.8) and (α=0.6, β=0.8). Their full widths at half maximum (FWHMs) are normalized by the emission wavelength λ. The measured FWHMs are 0.35 *λ* for the confocal case, 0.19 *λ* for (α=0, β=0.8), and 0.14 *λ* for (α=0.6, β=0.8). Notably, setting α=0 corresponds to a single subtraction $\left(I=I_{c}-\beta I_{o}\right)$, while tuning α (with the same β) provides additional resolution gains. Figure 2(b) shows cross-sectional intensity profiles, confirming that the super-resolution parameters yield narrower PSFs than the confocal reference. In Figure 2(c), a 2D map of SNR (in dB, via 10 log_10_) illustrates how varying α and β modulates noise performance. Figure 2(d) plots FWHM*/λ* over the same parameter space, revealing resolution trends. Finally, Figure 2(e) plots SNR versus FWHM*/λ*, highlighting a trade-off: as the FWHM decreases (improved resolution), SNR correspondingly declines.

**Fig. 3:**
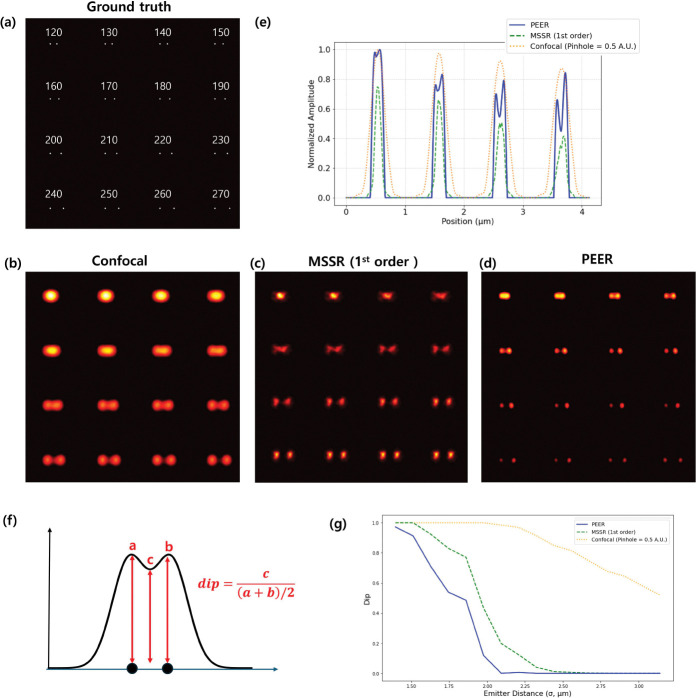
(a) Synthetic ground truth image with emitter pairs spaced from 120 nm to 270 nm; (b) confocal image (pinhole: 0.5 AU); (c) MSSR reconstruction (1st order); (d) PEER method reconstruction; (e) line profiles of the first row for confocal (orange dots), MSSR (green dashes), and the PEER method (blue line); (f) definition of the dip parameter; (g) dip results as a function of *σ* for the confocal PSF.

To further evaluate the lateral resolution of the novel method, simulations were conducted using synthetic images with predefined emitter distances. The emission wavelength was set to 680 nm in this simulation. The ground truth image (Figure 3(a)) comprises a pair of 40 nm beads spaced at intervals ranging from 120 nm to 270 nm. Figure 3(b) displays the simulated confocal image obtained by convolving the ground truth image with a PSF corresponding to a 0.5 AU pinhole size, where the PSF was derived from experimental measurements of the beads. In this confocal image, the pairs of beads closer than approximately 210 nm appear merged, revealing the practical resolution limit.

Figure 3(c) shows the result of applying the MSSR method (first-order implementation). While MSSR is also able to resolve closely spaced pairs down to 150 nm, the signal becomes more distorted as the emitter spacing decreases. Note that higher-order MSSR further enhances resolution but results in weaker signals. Details of the higher-order effects corresponding to this simulation can be found in Supplementary Figure 1. Figure 3(d) presents the PEER method, which also resolves closely spaced pairs down to 130 nm but exhibits fewer artifacts and stronger signals, indicating a more robust reconstruction compared to MSSR for highly confined emitters.

Figure 3(e) depicts line profiles for the first row (bead spacings of 120 nm to 150 nm), comparing confocal imaging, MSSR, and the PEER approach. Both MSSR and the PEER method deliver superior resolution relative to the confocal image.

To quantify these observations, the dip parameter (Dip) is introduced, as shown in Figure 3(f). Let a and b be the intensities of the two peaks, and c the intensity at the valley between them; then

$$\text{Dip}=\frac{2c}{a\;+\;b}$$

A lower Dip indicates better peak separation. Figure 3(g) plots Dip versus emitter distance, referencing σ as the standard deviation of the confocal PSF (approximated as Gaussian). Based on the Rayleigh criterion (Dip ≈ 0.8), the confocal image exhibits a rapid increase in Dip as the emitter distance approaches 2.5 *σ*, reflecting the theoretical resolution limit. For MSSR, it successfully resolves emitters separated by 150 nm, corresponding to 1.8 *σ*. The PEER method demonstrates the ability to resolve emitters at 1.6 *σ*. Regarding lateral resolution, the PEER method performs better than MSSR 1st order.

Collectively, these results illustrate that the PEER method and MSSR achieve comparable lateral resolution improvements over confocal imaging. However, when emitter spacing becomes very small, the PEER method displays less image distortion and stronger signals, suggesting that it is better suited for imaging scenarios where closely spaced structures must be resolved without significant artifacts.

**Fig. 4:**
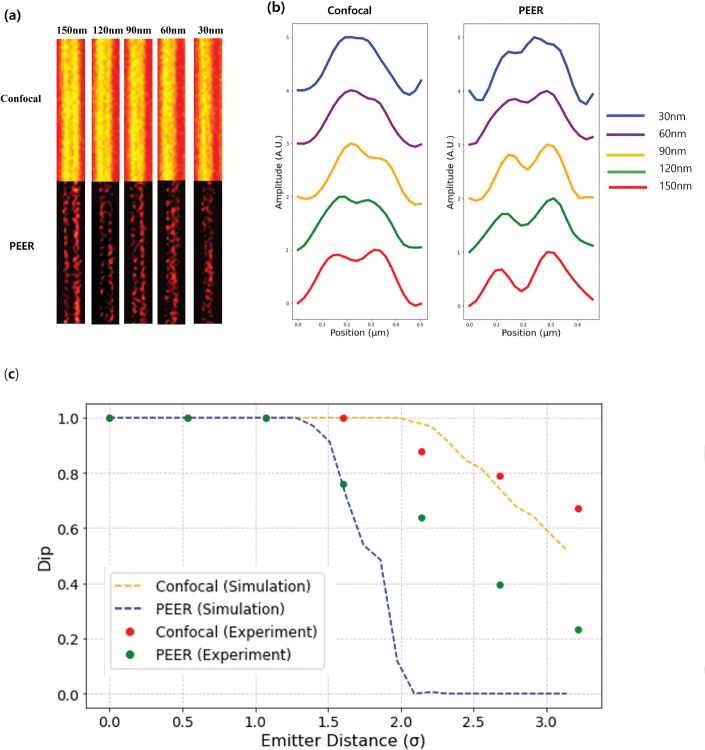
(a) Confocal (top) and PEER method (bottom) images for emitter spacings of 150, 120, 90, 60, and 30 nm, illustrating resolution improvements. (b) Line profiles (color-coded by spacing) comparing confocal imaging (left) and PEER method (right), further highlighting the enhanced resolving capability of the PEER approach. (c) Comparison of the measured Dip for Confocal and PEER in both simulation (dashed) and experiment (dots) as a function of the emitter distance *σ*.

To assess the lateral resolution of the PEER method, we utilized the ArgoLight test slide (Argo-SIM, pattern E), which contains progressively decreasing line spacings of fluorescent molecules. Imaging was performed using a 405 nm excitation laser, with emission collected at 450 nm. Figure 4(a) shows results for five representative line spacings: 150 nm, 120 nm, 90 nm, 60 nm, and 30 nm. The top row depicts confocal images acquired with a 0.5 AU pinhole, while the bottom row displays images reconstructed using the PEER method.

The theoretical diffraction limit for this confocal system is 130 nm when the emission wavelength *λ*_em_ = 450 nm, the numerical aperture NA = 1.4, and 0.5 AU.

In the confocal experimental data, the Rayleigh criterion is satisfied at a spacing of 150 nm, while line pairs at 120 nm and below appear merged, consistent with the theoretical resolution limit of the system.

In contrast, the PEER method significantly enhances resolution, resolving line patterns down to 90 nm, well below the Rayleigh criterion for confocal imaging. Notably, 90 nm corresponds to approximately 1.65 *σ*, where *σ* represents the standard deviation of the PSF. This observation agrees with simulations predicting resolution near 1.6 *σ*. However, at smaller spacings of 60 nm and 30 nm, the PEER method does not meet the Rayleigh criterion; while some degree of separation is discernible, noticeable merging artifacts indicate that these spacings fall below the method’s reliable resolution threshold.

Figure 4(b) compares the intensity line profiles for five different spacings in both the confocal and the PEER-method images. In the confocal profiles, line pairs at 150 nm and below exhibit broad peaks with minimal separation, emphasizing the system’s resolution limit. In contrast, the PEER method produces sharper peaks and deeper valleys, achieving distinct resolution down to 90 nm. At spacings of 60 nm and 30 nm, partial separation is observed; however, the peaks do not fully satisfy the Rayleigh criterion, highlighting the improved but finite resolving capability of the PEER method at these tighter spacings.

Comparison of the Dip measured for Confocal and PEER methods is shown in both simulation (dashed lines) and experiment (dots) as a function of the emitter distance (σ) in Figure 4(c). The orange and blue dashed lines represent the simulated Confocal and PEER data, respectively, while the red and green dots depict the corresponding experimental data. By interpolation, the emitter distances satisfying the Rayleigh criterion from the experimental data are found to be 1.5*σ* for PEER and 2.56*σ* for Confocal, in good agreement with the simulation. Beyond the Rayleigh limit discrepancies between the simulation and the experiment were observed for the confocal and PEER methods, which may be caused by underestimating the real noise scenario.

**Fig. 5:**
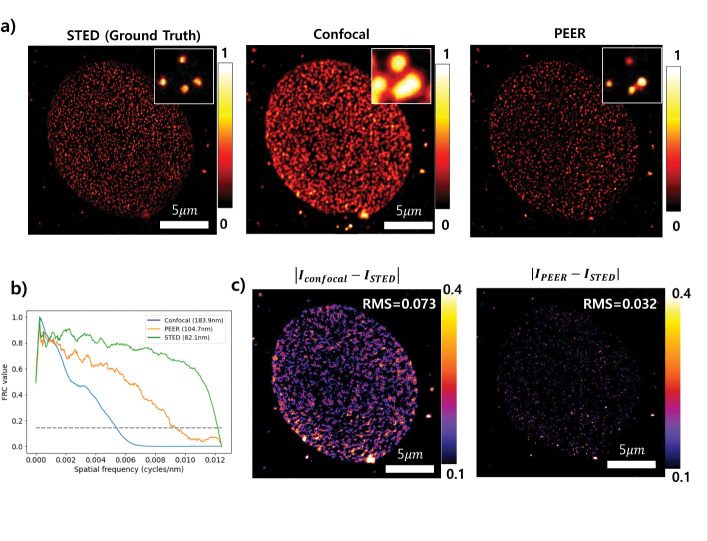
Validation of the PEER method. (a) Direct comparison of confocal and DTrCM with a ground-truth image STED image. (b) Resolution estimation using FRC of images showed in (a). (c) Absolute intensity difference between the ground truth image and both the diffraction-limited and SR images. Confocal and STED images were collected with a 10 μs pixel dwell time and two and six line accumulations, respectively. Sequential images were collected with a pinhole size of 0.59 AU and 1.83 AU at 650 nm. The depletion power was set to 17.091%. Pixel size: 40 nm.

An Abberior cell microscopy slide stained for the nuclear pore complex and Golgi proteins was used to validate the PEER method. Figure 5(a) presents a direct comparison of confocal and PEER images with a ground-truth STED image, with an inset illustrating a zoomed region for each image. Panel 5(b) shows the resolution estimation via Fourier ring correlation (FRC) [19], where the green, orange, and blue curves represent STED, PEER, and confocal, respectively. The measured resolutions from these curves were 82 nm, 104 nm, and 183 nm for STED, PEER, and confocal, respectively. Figure 5(c) shows the error map between the ground-truth image and both the confocal and PEER images. The inset shows the root mean square (RMS) value, being 0.073 and 0.032 for confocal and PEER, respectively. The lower RMS value of PEER indicates a greater similarity with the ground truth image (STED). Confocal and STED images were acquired with a 10 μs pixel dwell time and two and six line accumulations, respectively. Sequential images were recorded at pinhole sizes of 0.59AU and 1.83 AU for an emission wavelength of 650 nm, with the depletion power set to 17.091

### 2.2 Confocal sectioning depth enhnacement by pinhole engineering

**Fig. 6:**
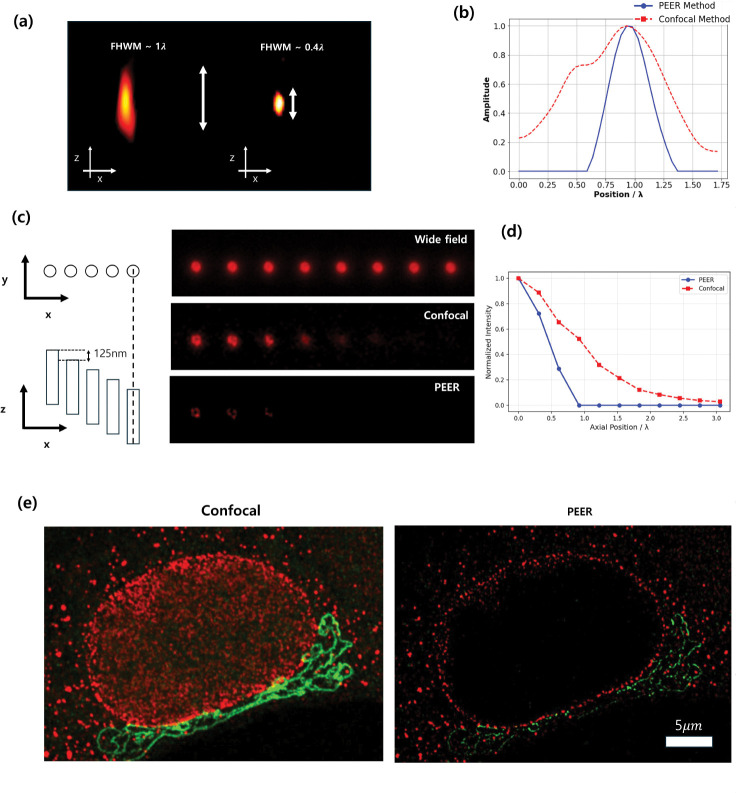
(a) experimental PSF of the confocal 0.5 AU (left) and the PEER method (right) in the xz domain. (b) Axial line profiles comparing the confocal 0.5 AU (blue) and the PEER method (orange). (c) Schematic of hollow cylindrical emitters spaced 3.6 μm laterally and embedded axially in 0.125 μm steps (left), with experimental results from widefield, the confocal 0.5 AU, and the PEER method images (right) using a 405 nm laser. (d) Normalized intensity profiles as a function of axial position (in units of *λ*) for the cylindrical emitters, measured by the PEER method (blue) and the confocal method (red), corresponding to the images in (c). (e) Optical sectioning enhancement for imaging NPC and Golgi structures in a fixed cell using PEER. Sequential confocal images were collected using a 32 μm (0.59 AU @ 650 nm) and a 100 μm (1.83 AU @ 650 nm) pinhole. Pixel size: 50 nm.

Figure 6(a) presents axial cross-sectional views of a 40nm bead measurement. In this panel, the left image shows the *xz*-plane PSF using a 0.5AU pinhole, whereas the right image illustrates the *xy*-plane PSF reconstructed with the PEER method. Figure 6(b) shows the corresponding line profiles along the *z*-axis, revealing the axial distributions achieved by each approach. The axial FWHM from the confocal measurement is approximately λ, while the FWHM with the PEER reconstruction is reduced to about 0.4 *λ*. This result closely matches the simulation Supplementary Figure.2 and verifies that the PEER method enhances optical sectioning by approximately 2.5-fold.

To validate the consistency of the PSF results, an additional experiment was performed using a three-dimensional fluorescent sample (Argolight 3D “crossing stairs” pattern), which consists of cylindrical structures embedded at various depths with a 0.125 nm periodicity (see figure 6(c)). Moving from left to right in the sample, each cylinder is positioned progressively deeper. Three imaging modes were compared:

1. Wide-field imaging, displayed as a *z*-projection of the entire stack.
2. Confocal imaging at 0.5 AU, where only the top slice of the confocal stack is shown.
3. The PEER method, which also uses the top slice from the confocal images acquired at 0.5 AU and 1.0 AU.

For the confocal data, only a single plane of the 3D stack are utilized, and the cylinders farther to the right, embedded deeper in the sample, appear increasingly out of focus. Compared to conventional confocal imaging, the PEER method exhibits enhanced lateral resolution and contrast, making the ring pattern more distinct. Additionally, while the confocal image reveals up to the sixth cylinder, the PEER method clearly resolves approximately three cylinders. This difference indicates that the PEER method has an effective imaging depth approximately 250 nm thinner than that achieved by conventional confocal imaging. Figure 6(d) shows the normalized intensity profiles, in units of axial position (*λ*), for the cylindrical emitters measured by the PEER method (blue) and the confocal method (red). According to the half-maximum intensity criterion, the PEER method achieves a sectioning depth of 0.46 *λ*, whereas the confocal approach extends to 0.98 *λ*.

Figure 6(e) shows the effect of optical sectioning enhancement in a biological specimen. The nuclear pore complex, inmunolabeled with abberior STAR RED (Ex/Em = 638/655 nm), is embedded in the nuclear membrane [20, 21], while the Golgi, inmunolabeled with abberior STAR Orange (Ex/Em = 589/616 nm), is located in the perinuclear region. While the long PSF of confocal imaging captures numerous structures, the far smaller PSF of PEER leads to signifcantly improved optical sectioning.

### 2.3 Super resolution fluorescence lifetime imaging by pinhole engineering

Monte Carlo simulations were conducted to assess the capability of the PEER method in resolving two fluorescent beads with distinct lifetimes. The beads were spaced 180 nm apart and emitted at 680 nm, and were modeled using a confocal PSF derived from experimental measurements with a 0.5 AU pinhole size. The left bead was assigned a fluorescence lifetime of 1 ns, while the right bead’s lifetime was 3 ns. Each bead had a radius of approximately 24 nm, and the total fluorescence intensity was limited to about 1000 photons per pixel. A background signal of about 10 photons per pixel was introduced to emulate realistic noise conditions.

**Fig. 7:**
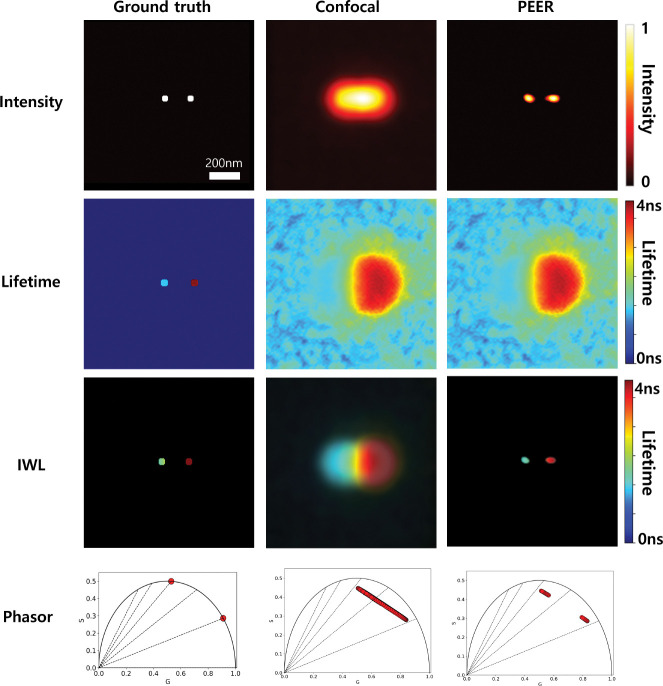
Monte-carlo simulation results for resolving two fluorescent beads spaced 180 nm apart, each with distinct lifetimes (1 ns and 3 ns) and emitting at 680 nm. The rows represent (top to bottom): intensity maps, lifetime maps, intensity-weighted lifetime maps (IWL), and phasor plots. Columns correspond to the ground truth, confocal imaging (0.5 AU pinhole), and the PEER method.

Figure 7 presents the simulation results. The rows display the intensity, fluorescence lifetime, intensity-weighted lifetime, and phasor plots, respectively. Each column corresponds to the ground truth, the confocal image, and the PEER method.

In the ground-truth intensity distribution, both beads are represented as distinct, sharp peaks. However, in the confocal image, the two beads merge into a single structure due to the resolution limit. In contrast, the PEER method successfully separates the two beads, accurately recovering the original spatial configuration in close agreement with the ground truth.

For the fluorescence lifetime maps, the ground truth accurately represents the distinct lifetimes of the two beads at 1 ns and 3 ns. While the fluorescence lifetime map measured by the confocal system shows significant merging and blurring of these lifetime signals, a gradient between the two beads remains, indicating that some spatial information for differentiation still exists. Notably, the same lifetime map was used in the PEER method.

The intensity-weighted lifetime maps further highlight the resolution enhancement from the PEER approach. While the ground-truth map shows two well-separated signals at 1 ns and 3 ns, the confocal image merges the signals into a blurred overlap. The PEER method, however, preserves the spatial fidelity of these signals, yielding a clear intensity-weighted lifetime map that distinctly separates the two beads.

Phasor analysis corroborates these findings. The ground-truth phasor plot displays two distinct points, corresponding to the 1 ns and 3 ns lifetimes of the beads. In the confocal phasor plot, these points merge into a single, elongated trajectory, reflecting overlapping lifetime components. In contrast, the PEER method reconstructs two separate phasor points, accurately distinguishing the differing lifetimes and spatially resolving the beads in the phasor space. This improvement arises because, although the confocal and PEER methods share the same *G* and *S* values, the intensity threshold applied in the PEER method allows for better differentiation.

Overall, these results demonstrate the ability of the PEER method to resolve sub-diffraction features while recovering distinct lifetime properties in demanding imaging contexts. By separating beads spaced at 180 nm and maintaining their individual fluorescence lifetimes, the PEER method transcends the resolution limits of conventional confocal imaging. Furthermore, the integration of lifetime and phasor information illustrates the robustness and potential of this approach for visualizing complex biological systems with high precision.

**Fig. 8:**
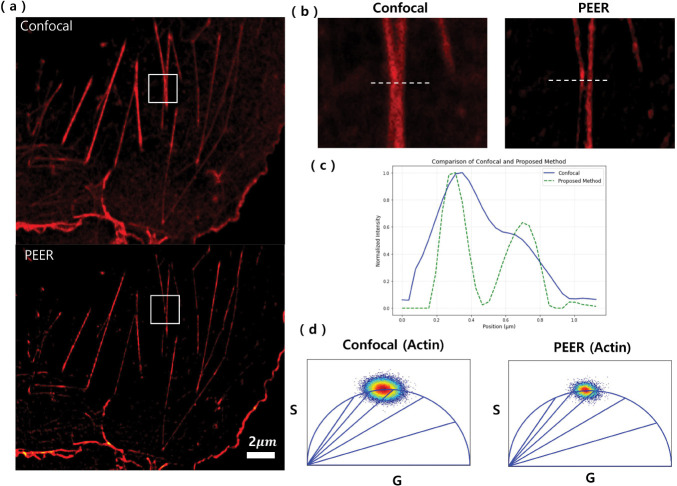
(a) Actin filament images in COS-7 cell line stained with Alexa Fluor^™^ 488 Phalloidin, acquired using confocal imaging (top) and the PEER method (bottom), highlighting enhanced contrast and resolution. (b) Magnified view of actin filaments with a dashed line marking the region used for generating intensity profiles. (c) Normalized intensity profiles of actin filaments along the dashed line in (b), comparing confocal imaging (blue line) and the PEER method (green dotted line). (d) Phasor distributions for actin filaments, demonstrating fluorescence lifetime characteristics.

Figure 8(a) presents actin filaments in COS-7 cells stained with Alexa Fluor 488 under confocal (top) and the DTrCM method (bottom) conditions. The enhancement in contrast in the SR images is attributed to its more effective rejection of out-of-focus light, providing clearer subcellular details. A close-up of the actin filaments is shown in Figure 8(b), with a dashed line indicating the region used for generating intensity line profiles. Figure 8(c) displays these normalized intensity profiles, where the peaks in the result of the introduced method appear sharper and the valleys are deeper. This reflects the improved lateral resolution, which makes individual filaments more distinct, and the reduced background from out-of-focus regions, highlighting the method’s superior resolution and contrast compared to confocal imaging. Finally, Figure 8(d) presents the phasor distributions for actin. While the PEER method presents enhanced spatial resolution, it does not significantly affect the intrinsic lifetime characteristics. Specifically, the standard deviation of the phasor distribution is 0.049 in the confocal data and 0.037 in the PEER method data. This reduction in standard deviation suggests that rejecting out-of-focus signals improves the precision of lifetime measurements. Collectively, these results demonstrate that the PEER method effectively enhances image clarity by minimizing out-of-focus contributions while preserving critical fluorescence-lifetime information. The validation of the time-resolved PEER method was also demonstrated in GFP-labeled mitochondria (see Supp fig. 3).

**Fig. 9:**
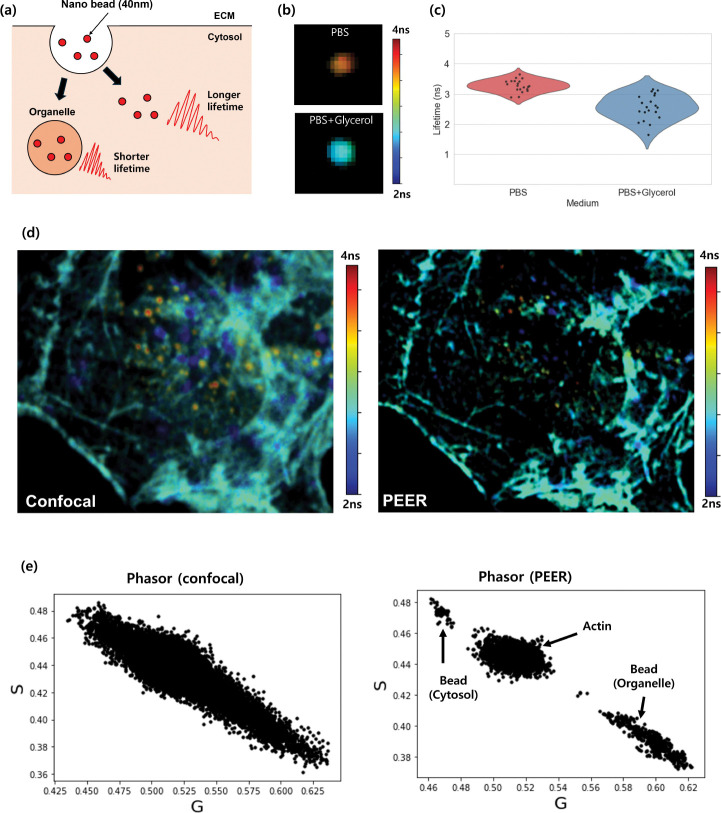
Nanobead uptake experiments. (a) Schematic for the endocytosis pathway of nanoparticles. (b) FLIM images of nanoparticles in PBS (refractive index 1.33) versus PBS with 50% glycerol (1.40). (c) Quantification of the lifetimes in PBS and PBS with glycerol (# of beads=20). (d) Comparison of conventional confocal FLIM (left) and the PEER super-resolution FLIM method (right). (e) Phasor plots from confocal FLIM (left) and super-resolution FLIM (right).

We performed nanobead uptake experiments to demonstrate how super-resolution FLIM enhances multiplexing capability. Figure 9(a) illustrates the endocytosis pathway [22, 23] of 40 nm nanoparticles, which move through endosomes before reaching lysosomes. As they transition from endosomes (refractive index :1.36 [24]) to lysosomes (refractive index 1.6 [25]), the fluorescence lifetime of the nanoparticles decreases. Figure 9(b) shows FLIM of nanoparticles in PBS (refractive index 1.33) and in PBS containing 50% glycerol (refractive index :1.40 [26]). The measured fluorescence lifetime decreases inversely with the square of the refractive index, showing the lifetime imaging dependancy to its local environment. Quantification of the lifetimes in these two media (Figure 9(c)) reveals a statistically significant reduction in the higher-index condition. Ten nanobeads were used to calculate the mean and standard deviation.

A comparison of conventional confocal FLIM (Figure 9 (d), left) and the PEER super-resolution FLIM approach (Figure 9(d), right) indicates that the actin filament network exhibits a relatively uniform fluorescence lifetime (2.8 ns). However, the nanoparticles show distinct short- and long-lifetime populations, reflecting different intracellular microenvironments, particularly varying refractive indices. After internalization, nanoparticles reside in endosomes with a refractive index (1.35) comparable to the cytosol, causing only a minor lifetime change (3.3 ns) relative to PBS (3.4 ns). By contrast, higher-index lysosomes reduce their lifetime more significantly. The super-resolution FLIM method thus enables clearer distinction among intracellular compartments.Figure 9(e) presents phasor plots of the FLIM data from the confocal (left) and super-resolution (right) methods. The confocal phasor plot shows a broad, overlapping distribution of lifetimes, obscuring the presence of multiple distinct components. In contrast, the super-resolution approach clearly separates nanoparticles located in different intracellular regions: actin filaments (middle cluster), nanoparticles in endosomes (top cluster), and nanoparticles in lysosomes (bottom cluster). These data confirm that the heterogeneous refractive indices in these organelles elicit distinct fluorescence lifetime shifts, showing the advantages of super-resolution FLIM for resolving multiplexed signals from individual nanoparticles in complex cellular environments. Despite the apparent change in the measured fluorescent lifetime value of the nanoparticle due to a change in the refractive index of the organelle, other possible alternatives, such as changes in pH and viscosity, should be further investigated.

## 3 Conclusion and discussion

This study demonstrates the efficacy of a super-resolution imaging method, leveraging pinhole engineering to enhance both lateral resolution and depth sectioning compared to conventional confocal imaging. The enhancement in lateral resolution is evident from both simulation and experimental data. Simulations indicate that the PEER method can resolve emitter pairs at approximately 1.6 *σ*, a limit validated using a super-resolution calibration target. Experimentally, the PEER method resolves features down to 90 nm, surpassing the theoretical Rayleigh criterion for confocal systems (150 nm) with 405 nm excitation. Furthermore, simulations confirm the method’s ability to resolve closely spaced structures without significant distortion or artifacts, a critical capability for sub-diffraction fluorescence imaging. Beyond lateral resolution, the method’s ability to improve axial sectioning was demonstrated with three-dimensional samples. The improved axial sectioning capability (250 nm thinner than confocal imaging) underscores the method’s superior rejection of out-of-focus signals, resulting in sharper and more precise visualization of subcellular structures. This enhanced contrast enables clearer identification of features such as actin filaments and mitochondrial networks.

Phasor-based lifetime imaging further highlights the robustness and versatility of the PEER approach. While preserving the intrinsic fluorescence lifetime characteristics of the samples, the method enhances the spatial resolution of lifetime maps and reduces variability in lifetime measurements, as evidenced by a lower standard deviation in the phasor distributions. This capability is particularly valuable for applications requiring high-precision spatial and lifetime information, such as the study of subcellular structures. Notably, fluorescence lifetime imaging is a highly promising tool for NADH and FAD metabolic imaging, and this super-resolution technique holds significant potential in advancing these applications. One critical application is mitochondria-specific NADH imaging. Since mitochondria are often smaller than the diffraction limit, localizing their precise position poses a challenge for conventional confocal microscopy. However, this approach achieves a lateral resolution of 100 nm and enhanced axial sectioning, making mitochondria-specific metabolic imaging both feasible and highly precise.

FLIM has been used in confocal and STED imaging to remove undesired backscattered light and early photons, improving effective spatial resolution. Here, the benefits of SR in improving the precision of lifetime measurements are also demonstrated (see Figure 7(e)). Recently, a multicolor super-resolution imaging method called phasor-based fluorescence spatiotemporal modulation was developed, requiring two beams with Gaussian and donut-shaped modes [27]. In contrast, our technique does not require system modifications, and can be implemented in any commercial confocal FLIM setup, which serves as a strong advantage. In this work, a single detector with a motorized pinhole was used to facilitate spatial alignment. An efficient PEER implementation can be achieved using single-photon avalanche detector arrays to accelerate acquisition time for real-time applications, enabling the simultaneous collection of signals equivalent to both a closed and an open pinhole (see Suppl. Fig 4). In fact, our approach could benefit from the development of detector arrays based on inner and outer concentric sensors, which may simplify data handling for conventional sensor arrays and enhance signal detection by increasing the fill factor.

Overall, our method provides a cost-effective and hardware-independent solution for achieving super-resolution imaging with enhanced lateral and axial performance. It addresses critical challenges in fluorescence imaging, such as resolution limits and background noise, while preserving key fluorescence-lifetime information. These attributes position the method as a promising tool for advanced biological imaging applications, enabling more detailed and accurate visualization of subcellular and molecular structures.

## 4 Methods

### 4.1 Experimental setup and data analysis

STED and confocal images were imaged using a 100×/1.45 oil immersion objective lens with the Abberior STEDYCON module on a Nikon Eclipse Ti2-E wide-field microscope. Excitation at 561 and 640 nm was used, along with a 775 nm STED laser that was turned on/off depending on the image acquisition mode.

Confocal fluorescence lifetime images were captured using a custom-developed microscope (Intek Scientific, iRT-FLIM-001) described previously [17]. This system employs a picosecond laser (Prima, PicoQuant) operating at wavelengths of 450, 520, and 640 nm with a 25 MHz repetition rate, along with a nanosecond laser at 405 nm (Thorlab, NPL41B) and 488 nm (Thorlab, NPL49B) with a 10 MHz repetition rate. A dichroic mirror reflected the collimated pulsed laser beam, passed through an x-y galvanometer scanner (Thorlabs, model GVS002), and directed through optical components, including a 60x objective lens (Olympus, UPLXAPO60), for excitation and signal collection. The fluorescence signal from the sample was transmitted through the dichroic mirror and further filtered by a bandpass filter in the spectral range of 415–450 nm for 405 nm excitation (Semrock, FF01–450 / 70–25), 510–550 nm for 488 nm excitation (Semrock, FF01–535 / 50–25) and 660–700 nm for 640 nm excitation (Semrock, FF01–679 / 41–25). Subsequently, the filtered fluorescence signal was focused by a lens, passed through a motorized pinhole, and collected by a photomultiplier tube (PMT, Hamamatsu, H10720–01). Sequential images were acquired with an open and closed pinhole to reconstruct the SR PEER image following Eq. (3).

The acquired time-resolved digitized images were processed in parallel using a commercial FLIM software package (Intek Scientific), leveraging GPU processing with the CUDA architecture for phasor analysis. The g and s values were calculated in real-time using cosine and sine transforms, implemented with fast Fourier transforms for dual modulation frequencies at 50 MHz and 100 MHz. For the data analysis, a commercial FLIM software tool (LifetimeXplorer v1.10.9 by Intek Scientific) was employed to conduct the initial phasor analysis of the acquired data. This platform applies real-time fast Fourier transforms (FFTs) at two modulation frequencies, 50 MHz and 100 MHz. Let f denote the modulation frequency and F the fluorescence decay signal. The angular frequency is then given by:

ω=2πf.

The dimensionless phasor coordinates, G and S, are defined as follows:

$$G=\frac{\int Fcos(\omega t)\;dt}{\int F\;dt},\;S=\frac{\int F\;sin(\omega t)\;dt}{\int F\;dt}.$$

For the lifetime map, the *phase lifetime* was used, which can be calculated as:

$$\tau_{phase}=\frac{S}{G\omega }.$$

### 4.2 Sample preparation

A two-color cell slide (IG2COLOR-4006, Abberior, Germany) was used, containing fixed mammalian cells immunostained for Nuclear Pore Complex (NPC) proteins labeled with Abberior STAR RED and Golgi proteins labeled with Abberior STAR ORANGE. The cell specimen was mounted on a glass slide using Abberior Mount Solid Antifade.

COS-7 cells stained with Alexa Fluor^™^ 488 Phalloidin: COS-7 cells were cultured in DMEM media (Corning^™^ DMEM with L-Glutamine, 4.5 g/L Glucose and Sodium Pyruvate, cat. no. 10013CV) supplemented with 10% fetal bovine serum (FBS) (Corning cat. no. 35011CV) and 100 IU/mL penicillin and 100 μg/mL (Corning). Cells were plated onto glass coverslips (ZEISS, thickness no. 1 ½ high-performance 18 mm × 18 mm, 0,170,+/− 0,005 mm) and incubated for 24 hours. Cells were washed 3x with phosphate buffered saline (PBS) and fixed using 4% paraformaldehyde (EMS, Formaldehyde Aqueous Solution (Paraformaldehyde Aqueous Solution) EM Grade)) for 10 min. Cells were washed 3X with PBS and cells were extracted by incubating in 0.1% Triton-X 100 (Sigma, cat. no. T9284–500mL) for 10 min. Cells were rinsed 3x with PBS and unspecific binding was blocked by incubating cells for 30 min in Phosphate Buffered Saline with 0.1% Tween 20 (Sigma: cat. no. P9416–100ML) (PBST). Next, PBST was removed and a 40x dilution of Alexa Fluor^™^ 488 Phalloidin (Thermo Fisher, cat. no. A12379) was prepared in PBS with 1% Bovine Serum Albumin (BSA) and added directly onto the cells on the coverslips and incubated for 20 min in the dark at room temperature. Alexa Fluor 488 phalloidin solution was removed and cells were rinsed 1x with PBS. Finally, coverslips were mounted onto glass slides with mounting medium (Abberior, Abberior mount, Liquid antifade, cat. no. mm-2009–2×15ML) and sealed with clear nail-polish.

Cellular uptake of 40 nm beads: COS-7 cells were cultured in DMEM media (Corning^™^ DMEM with L-Glutamine, 4.5 g/L Glucose and Sodium Pyruvate, cat. no. 10013CV)) supplemented with 10% fetal bovine serum (FBS) (Corning cat. no. 35011CV) and 100 IU/mL penicillin and 100 μg/mL streptomycin (Corning, cat. no. MT-30–001-CI) in a humidified 5% CO_2_ balanced-air atmosphere at 37 °C. Cells were plated onto glass coverslips (ZEISS, thickness no. 1 ½ high-performance 18 mm × 18 mm, 0,170,+/− 0,005 mm) and incubated for 24 hours. 40 nm STED Beads (Abberior, 20x) were added to the cells using a 15x dilution of the 20x stock solution for a total dilution of 300x in 1 mL of media and incubated for 24 hours. Media was removed, and cells were washed 3x with phosphate buffered saline (PBS) and fixed using 4% paraformaldehyde (EMS, Formaldehyde Aqueous Solution (Paraformaldehyde Aqueous Solution) EM Grade)) for 10 min. Cells were washed 3X with PBS and cells were extracted by incubating in 0.1% Triton-X 100 (Sigma, cat. no. T9284–500mL) for 10 min. Cells were rinsed 3X with PBS and unspecific binding was blocked by incubating cells for 30 min in Phosphate Buffered Saline with 0.1% 0.1% Tween 20 (Sigma: cat. no. P9416–100ML) (PBST). PBST was removed and phalloidin staining solution (Abberior, phalloidin STAR red) was added onto the coverslips at a concentration of 1Unit/ml in PBST of the stock solution (200 units/ml in DMSO) and incubated for 1 hour in a humidity chamber. Staining solution was removed, and cells were rinse in PBS 1x. Coverslips were mounted onto glass slides with mounting medium (Abberior, Abberior mount, Liquid antifade, cat. no. mm-2009–2×15ML), and sealed with clear nail-polish.

## Supplementary Material

Supplementary Files

This is a list of supplementary files associated with this preprint. Click to download.

• SuperresolutionFLIMSupple1.pdf
